# The Pooled Diagnostic Accuracy of Neuroimaging, General Movements, and Neurological Examination for Diagnosing Cerebral Palsy Early in High-Risk Infants: A Case Control Study

**DOI:** 10.3390/jcm8111879

**Published:** 2019-11-05

**Authors:** Catherine Morgan, Domenico M. Romeo, Olena Chorna, Iona Novak, Claire Galea, Sabrina Del Secco, Andrea Guzzetta

**Affiliations:** 1 Cerebral Palsy Alliance Research Institute, Child and Adolescent Health, The University of Sydney, Sydney, NSW 2050, Australia; INovak@cerebralpalsy.org.au (I.N.); cgalea@cerebralpalsy.org.au (C.G.); 2 Pediatric Neurology Unit, Fondazione Policlinico Universitario A. Gemelli, IRCCS, 56128 Rome, Italy; domenicomarco.romeo@policlinicogemelli.it; 3 Department of Developmental Neuroscience, Stella Maris Scientific Institute, 00168 Pisa, Italy; ochorna@fsm.unipi.it (O.C.); sabrina.delsecco@gmail.com (S.D.S.); aguzzetta@fsm.unipi.it (A.G.); 4 Grace Centre for Newborn Care, Children’s Hospital at Westmead, Sydney, NSW 2145, Australia; 5 Department of Clinical and Experimental Medicine, University of Pisa, 56126 Pisa, Italy

**Keywords:** cerebral palsy, diagnostic accuracy, general movements, HINE

## Abstract

Introduction: Clinical guidelines recommend using neuroimaging, Prechtls’ General Movements Assessment (GMA), and Hammersmith Infant Neurological Examination (HINE) to diagnose cerebral palsy (CP) in infancy. Previous studies provided excellent sensitivity and specificity for each test in isolation, but no study has examined the pooled predictive power for early diagnosis. Methods: We performed a retrospective case-control study of 441 high-risk infants born between 2003 and 2014, from three Italian hospitals. Infants with either a normal outcome, mild disability, or CP at two years, were matched for birth year, gender, and gestational age. Three-month HINE, GMA, and neuroimaging were retrieved from medical records. Logistic regression was conducted with log-likelihood and used to determine the model fit and Area Under the Curve (AUC) for accuracy. Results: Sensitivity and specificity for detecting CP were 88% and 62% for three-month HINE, 95% and 97% for absent fidgety GMs, and 79% and 99% for neuroimaging. The combined predictive power of all three assessments gave sensitivity and specificity values of 97.86% and 99.22% (PPV 98.56%, NPV 98.84%). Conclusion: CP can be accurately detected in high-risk infants when these test findings triangulate. Clinical implementation of these tools is likely to reduce the average age when CP is diagnosed, and intervention is started.

## 1. Introduction

Cerebral palsy (CP) identifies a group of developmental disorders of movement and posture, causing activity limitation, attributed to non-progressive disturbances related to brain injury early in development [1,2]. It is considered one of the most common causes of childhood physical disability with an overall prevalence of 2.11 per 1000 live births [3], with the prevalence starting to decline by 30% in high-income countries [4].

Establishing an early diagnosis of CP is important as this can expedite early intervention, thereby maximizing the opportunities for appropriate physical and learning support, optimizing neuroplasticity, prevention of complications, and provision of adequate psychological support to parents. A recent International Clinical Guideline for the early identification of CP recommends the use of neuroimaging, Prechtls’ General Movements Assessment (GMA), and the Hammersmith Infant Neurological Examination (HINE) for early detection of CP [5,6,7,8]. Each of these assessments has been rigorously tested in high-risk infant populations demonstrating high sensitivity and specificity for detecting CP as early as three months corrected age.

The Hammersmith Infant Neurological Examination (HINE), is a simple and scorable method designed for evaluating infants between 2 months and 24 months of age [6]. It includes 26 items that assess different aspects of neurological function, such as cranial nerves, posture, movements, tone, and reflexes. The HINE is easily performed and accessible to all clinicians; it can be completed in 5 to 10 min. A good interobserver reliability has been reported, even in inexperienced staff [6]. An optimality score was developed for research purposes. The optimality score is based on the frequency distribution of the scores in the normal population, defining optimal as all the scores found in at least 90% of a cohort of low-risk, typically developing infants assessed at different ages [9]. Each item is scored separately, from zero to three, and the individual scores are added to achieve a global optimality score. The global score can range from a minimum of 0 (if all the items score 0) to a maximum score of 78 (if each item scores three). Global scores are reported as optimal if they are equal or above 73 at 9 to 12 months, or equal or above 70 and 67 at 6 months and 3 months, respectively. Multiple studies in high-risk cohorts have been published and include helpful cut off scores for predicting CP during the first year of life [10]. A cut off score of 57 at age three months is recommended in the international guidelines, based on a large cohort study [5,10].

General movements (GMs) are part of the spontaneous movement repertoire, are present from early fetal life onwards until the end of the first half a year of life, and were first categorized by Heinz Prechtl [7,11]. GMs are complex, occur frequently, and last long enough to be observed properly. They involve the whole body in a variable sequence of arm, leg, neck, and trunk movements. They wax and wane in intensity, force, and speed, and they have a gradual beginning and end. Rotations along the axis of the limbs and slight changes in the direction of movements make them fluent and elegant and create the impression of complexity and variability. Two distinct GM patterns can be observed from term age: writhing movements and fidgety movements. Writhing movements are present from term age up to about nine weeks. Fidgety movements appear at approximately 7–8 weeks and can be present up to about 20 weeks when goal-directed movements start to prevail. If the nervous system is impaired, GMs lose their complex and variable character and become monotonous and poor.

During the writhing period abnormal GMs are described as either: (a) poor repertoire (PR) GMs, where the sequence of successive movement components is monotonous, and arm, leg, trunk, and head movements do not occur in the normal rich and complex sequence; (b) cramped-synchronized (CS) GMs, where the GMs appear rigid and stiff and lack the normal, smooth, and fluent character, and all the limb and trunk muscles contract and relax almost simultaneously; (c) chaotic (Ch) GMs where all limb movements are of large amplitude, occur chaotically, and lack fluency or smoothness, or both.

During the fidgety period, normal FMs are defined as circular movements of small amplitude, moderate speed, and variable acceleration of neck, trunk, and limbs in all directions. Abnormal GMs at the fidgety age are classified as follows: (a) absent FMs, when normal FMs are never observed from age 6 to 20 weeks’ post term age (PTA); (b) abnormal FMs, when FMs can be detected but their amplitude, speed, and jerkiness are moderately or greatly exaggerated. Cramped synchronized GMs in the writhing period and an absence of fidgety GMs in the fidgety period are predictive of CP with high sensitivity and have specificity values repeatedly demonstrated across multiple studies [12,13]. Other abnormal GM patterns, including poor repertoire and abnormal fidgety, are less predictive although are moderately correlated to various suboptimal developmental outcomes [14].

HINE, GMs, and neuroimaging address different, although overlapping, aspects of the condition, such as (i) pathogenesis, mainly targeted by the assessment of neuroimaging findings, (ii) impairment, especially addressed by neurological examinations like the HINE exploring posture, tone, and reflexes, and (iii) functional limitations, as expressed by abnormalities of neurodevelopment, as measured by the quality of movement [15]. Only a few studies have examined the predictive power of combining two of the three recommended assessments. One study of 86 preterm infants demonstrated that the GMA at three months and increasing severity of white matter injury on magnetic resonance imaging (MRI) accurately predicted CP [16]. Similarly, in a cohort of infants with perinatal asphyxia, cramped synchronized GMs, basal ganglia, and thalami involvement on MRI were found to highly correlate with a CP outcome [17]. In a large cohort of preterm infants [18], the integrated use of standardized neurological and motor assessment demonstrated a high correlation between low HINE scores, abnormal GMs at three months, and a later diagnosis of CP. To date, no study has examined the pooled predictive power of GMA, MRI, and HINE in the early prediction of CP for diagnostic or prognostic purposes. Increased accuracy of early detection could enable more timely access to early intervention and potentially to enable early treatments that minimize the development of secondary impairments. The aim of the present study is, therefore, to determine via a retrospective, case-control study, the pooled predictive power of these early assessment tools in CP in a population of high-risk infants.

## 2. Methods

We performed a retrospective case-control study of infants born in three hospitals in Italy (Catania, Rome, Pisa) between 2002 and 2016. Clinical data from infants with typical development, mild disability, or CP diagnosis at two years were included (Table 1). The mild disability group included children with a developmental quotient at 2 years or later below 85, but without a diagnosis of CP.

In all, gestation age at birth, birth weight, sex, neurological examination (HINE) score, GMA spontaneous movements assessments, neuroimaging data (MRI or Ultrasound), Gross Motor Function Classification System (GMFCS) in children with CP, and 24-month developmental quotient (DQ) were retrieved from medical records. 

Minimum required data for inclusion were gestation age at birth, date of birth, sex, HINE at 3 months, both writhing and fidgety GMA results, and 2-year outcome, including GMFCS level in children with a diagnosis of CP. We did not include records from infants with a history of congenital anomalies. 

Age correction was used, as per standard clinical protocol, for all evaluation and outcome data. For each group, infants’ data were matched for birth year (±1 year), gender, and gestational age at birth (by range of completed weeks at birth <28, 28–31, 32–36). A total of 720 records were evaluated, and 441 (147 in each group) were included.

General Movements: We retrieved the results of GMA, which were completed as a standard of care at two time points. Writhing and fidgety periods classifications were retrieved, as previously completed according to standard Prechtls’ spontaneous movements GMA method [19]. In all centres, GMs were scored by certified assessors who passed the Advanced GMs Course, thus warranting a high interscorer agreement. As part of the follow-up program, video recordings are performed during the hospitalisation and every 3–4 weeks in the outpatient clinic in all subjects. The video-camera was positioned approximately 1 m above the infant, at an angle of 45°. The infants were recorded during inter-feeding time, in a supine position, naked, or in a nappy. When more than 1 record of GMA classification was available, for the writhing period, the latest result was retrieved, and for the fidgety period, the one closest to 12 weeks post-term was retrieved.

Neurologic Examination: Hammersmith Infant Neurological Examination scores were retrieved from infants’ clinical records. All infants included in the study were assessed with the HINE at 3, 6, 9, and 12 months PTA. As the aim of the present work was to compare HINE and the other techniques, only the assessment performed at 3 months was considered for the study. In all centres, HINE was scored by trained assessors who have contributed to the development of the scale (AG, DR) and have shown high inter-scorer reliability in previous prospective studies [18].

Ethics: Since this is a retrospective study, no specific consent was obtained from the parents of the infants to retrieve the clinical information. Consent for video recording GMs was obtained from parents at the time of clinical assessments as per standard procedures at each of the centres. 

### 2.1. Outcome

Gross Motor Function Classification Scale (GMFCS) scores were retrieved from the medical record. Developmental quotient was derived from CAT-CLAMs (*N* = 182) or Griffiths (*N* = 118) assessments during the 24-month (±1 month) assessment visit. These tools were chosen as they are the ones routinely used for the clinical follow up at the participating centres. They both showed a high correlation with the Bayley Scales of Infant Development [20,21]. For outcome at two years, typical development was defined as a psychomotor developmental index (PDI) of ≥85.

Data on GMA at both writhing and fidgety age and HINE at 3 months was available in 100% of cases. Assessment results for all groups are summarised in Table 2.

### 2.2. Neuroimaging

MRI or ultrasound neuroimaging data obtained during the first semester of post-term life were derived from the medical records. MRI and or brain ultrasound reports were analysed by two independent scorers (AG and DR), blinded to any other clinical information and group allocation. When reports from both imaging modalities were available, they were both considered for the analysis. Scorers assigned each patient to one of the following groups, based on current knowledge on the predictive value of early neuroimaging in infants at-risk to develop CP [22,23]:

(i) No CP, i.e., extremely unlikely to develop CP. Children included in this group were typically those with normal imaging or with anomalies usually not associated with the development of CP, such as IVH grade 1 or transient flares

(ii) Unclear, i.e., when CP was possible but not very likely. Children included in this group were those with evidence of brain damage that, however, appeared to spare the motor structures. Typical examples are children with IVH, stroke, or hypoxic-ischaemic encephalopathy showing ischemic or haemorrhagic lesions not involving the perirolandic area, the Posterior Limb of the Internal Capsule (PLIC), the pyramids, or the basal ganglia.

(iii) CP, i.e., very likely to develop CP. Children included in this group were those with evidence of brain damage involving the motor structures. Typical examples were IVH grade IV, cystic PVL, HIE with BG involvement, or brain malformation with involvement of the motor cortex. 

Children were assigned to a different group by the two scorers, data were re-analysed, and a consensus was reached.

### 2.3. Statistical Methods

Frequency and percentage (%) were reported for categorical variables, and the mean and standard deviation (SD) were reported for continuous variables overall and for the three groups (CP, mild disability and controls). The median and interquartile range (IQR) were reported for the HINE for clinical interpretation. Chi-squared were investigated for associations between type of CP (Control + Mild versus CP) and General Movements (GMs), HINE, and MRI as categorical variables. 

Logistic regression was undertaken with outcome variables as no CP (control + mild disability) versus CP and the HINE at 3 months to determine the cut off value using sensitivity and specificity. Logistic regression was undertaken with outcome variable as CP versus no CP and the following predictor variables: HINE 3 months continuous, HINE 3 months dichotomised (less than 57 versus 57 or more); fidgety dichotomised (fidgety versus absent/abnormal), imaging ordinal (no CP, unclear, CP). Multiple logistic regression was then undertaken with outcome variable as no CP versus CP and variables: (a) HINE 3 months continuous + fidgety dichotomised, (b) HINE 3 months continuous + imaging ordinal, (c) fidgety dichotomised + imaging ordinal, (f) HINE 3 months continuous + fidgety dichotomised + imaging ordinal. Cases with abnormal fidgety movements (*n* = 42) were then removed and an analysis of fidgety (*n* = 258) versus absent fidgety (*n* = 141) was conducted. In the final multiple regression, we analysed the combined predictive value of HINE at 3 months + fidgety (absent versus fidgety) + imaging.

Akaike information criterion (AIC) was used to perform model comparisons. Area under the curve (AUC) for the Receiver Operating Characteristic (ROC) Curve was used to determine the accuracy of the test to correctly classify those with and without the outcome. The significance level was set at *p* < 0.05. All analysis was performed using STATA (STATA, Version 14, StataCorp, College Station, TX, USA).

## 3. Results

In this study, we purposively included and matched: 147 children with a CP diagnosis; 147 with a diagnosis of mild disability (not CP); and 147 children assessed to have a normal outcome. Of the 441 children, 221 were girls with a mean gestational age of 33.8 weeks and mean birth weight of 2.14 kg (Table 1). Three per cent of the birthweight data was missing from the charts. MRIs were obtained in 39 cases and brain ultrasounds in 437 cases (both were available in 35 cases). Children were assigned to a different group by the two scorers in 19 cases (Cohen’s K 0.91) before data were re-analysed, and a consensus was reached.

DQs were only available for 68% of the sample overall. While 76% of data was retrievable for the children with a mild disability, only 56% of the children with a CP diagnosis had a DQ calculated. In 71% of the children with a normal outcome DQ was reported, although in the majority of the cases simply as “higher than 85”, without specification on the actual score. A significant proportion of the children with CP were unable to be assessed on these norm-referenced measures due to the motor requirements of the cognitive tasks.

### 3.1. Infants without Disability

In children with a normal outcome (DQ > 85 at two years), the GMA at writhing age were normal in 82% of the sample, while the remaining infants were classified as poor repertoire. At fidgety age, all infants without disability had normal GMs. The median HINE score at 3 months was 63 (IQR 56–66) and at 12 months was 72 (IQR 70–74). In 73% of cases, neuroimaging accurately predicted a normal motor outcome; however, the prediction of CP was unclear in 26% of cases. Only 2 of the 147 children in this group were predicted by neuroimaging to have CP.

### 3.2. Infants with Cerebral Palsy

**Figure 1:**
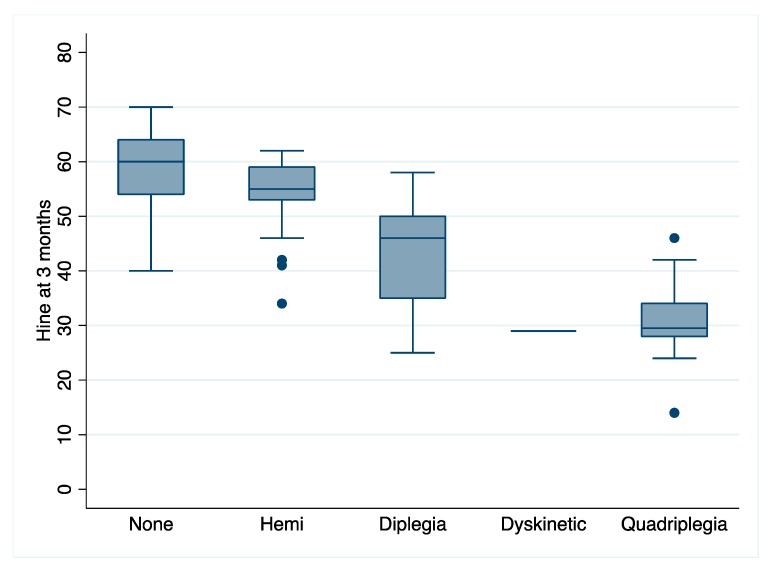
Hammersmith Infant Neurological Examination (HINE) scores across cerebral palsy (CP) type and topography.

Children with CP for whom data was available had a mean DQ of 77 (SD 17.85). GMFCS data for this group are summarised in Table 1. Only one infant had normal GMs during the writhing period, and 95% had abnormal GMs at 12 weeks. Of these, 96% were scored as absent fidgety. At three months, infants with CP had a median HINE score of 46 (IQR 34–55) and at 12 months was 59 (IQR 42–56). HINE data at three months were further analysed according to type/topography of CP at age 2, and this data is represented in Figure 1. There were 55 children with unilateral CP and the rest with bilateral (one dyskinesia and 36 with spastic quadriplegia). Almost 80% of infants later diagnosed with CP were predicted to have this diagnosis from neuroimaging alone; however, the score was unclear in 18%. Only 3 of the 147 children in this group were not predicted from imaging to have CP.

### 3.3. Children with a Mild Disability

Of the children with a non-CP disability, the mean DQ was 61 (SD 11.09), indicating most of these children had a mild to moderate intellectual disability. The distribution of GMs scores was more variable in this group with just over half of the group recording poor repertoire GMs around the equivalent term age. At fidgety age, just over 70% had normal fidgety GMs while 24% scored abnormal fidgety. Seven infants scored absent fidgety. Most infants (82%) were accurately predicted from neuroimaging not to have CP in this group with an unclear outcome prediction in only 16%.

### 3.4. Overall Accuracy of the Model

**Figure 2:**
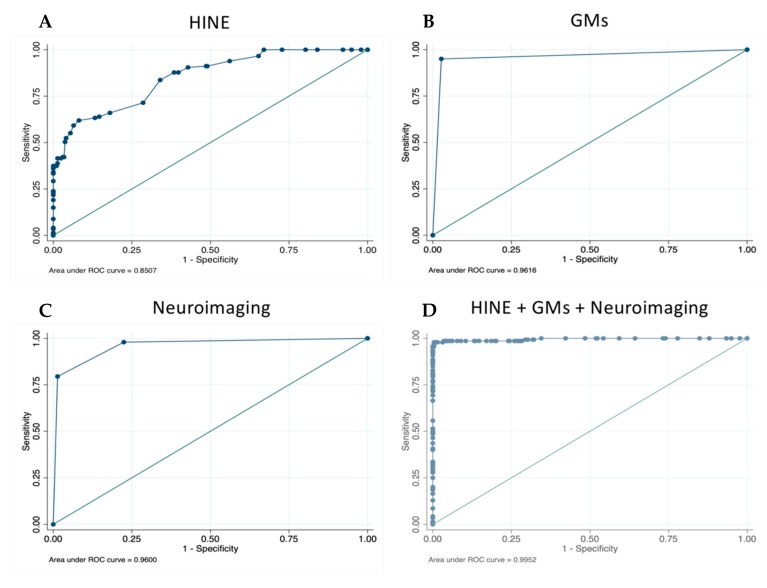
(**A**) Logistic regression of HINE at 3 months; (**B**) GMs, absent fidgety vs normal fidgety (abnormal removed); (**C**) neuroimaging, predict CP vs. predict no CP; (**D**) HINE + GMs (fidgety) + neuroimaging.

Each assessment (GMA, imaging and HINE) independently demonstrated excellent predictive validity for CP (Figure 2A–C; Table 3). GMA at fidgety age (absent fidgety vs normal) had the highest accuracy with 96.49% of infants correctly classified (sensitivity 95.04; specificity 97.29; PPV 95.04; NPV 97.29). This value was lower when abnormal fidgety was included in the model, with 88.66% of infants correctly classified. Neuroimaging independently classified 92.27% of children correctly as having CP. Sensitivity was 79.45, specificity 98.64 (PPV 96.67; NPV 90.63). HINE scores at 3 months gave a sensitivity score of 59.18, and specificity of 93.54 (PPV 82.08; NPV 82.09). Infants correctly classified using HINE alone was 82.09%.

When GMA and HINE scores at 3 months, plus early neuroimaging, were combined in the model, 98.74% of children were correctly classified (Figure 2D). Sensitivity for detecting CP was 97.86%, and specificity was 99.22% (PPV 98.56; NPV 98.84).

## 4. Discussion

We sought to calculate the pooled predictive power of early GMA, neuroimaging, plus HINE in diagnosing CP in a population of high-risk infants via a retrospective case-control study. We found that the pooled predictive power of early GMA, neuroimaging, plus HINE was highly accurate, with a sensitivity for detecting CP of > 97% and a specificity of > 97%. The pooled sensitivity and specificity of the three instruments together was higher than the three tools in isolation, where literature reports the GMA has a sensitivity of 98%, neuroimaging (i.e., MRI) has a sensitivity of 86–89%, and the HINE has a sensitivity of 90% [5]. The advantage of using three instruments together is that each tool measures a different but complementary construct: MRI measures neuroanatomy detecting brain abnormalities, HINE measures neurological function detecting disorders of posture, and GMA measures the quality of movement detecting disorders of movement. It is worth noting that all the measures had good sensitivity in isolation, especially absent fidgety GMA, but the advantage of combining tests was to increase the predictive power for CP since all three tests also predict other neurological disorders that are not CP. In some settings, all three tests may not be clinically feasible, and in these circumstances, two tests are better than one to increase clinical certainty.

Our findings are, therefore, clinically important because the clinical diagnosis of CP has traditionally been made late at 12–24 months of age [24] because of the lack of biomarkers. Historically, the diagnosis might be suspected but is often delayed until the clinician is more certain, thereby delaying the difficult conversation with parents of giving a complex and potentially stigmatising diagnosis. Advancement towards accurate early diagnosis has been made in international clinical guidelines, where it is recommended that clinicians use a combination of standardised tests (GMA, MRI, and HINE) [5], rather than relying on history taking and clinical observations, which can be falsely reassuring with late-onset spasticity or when milestones are initially achieved on time. This is the first study to analyse the pooled predictive power of the three tests, in an effort to improve clinician confidence in the accuracy of an early diagnosis and thereby bring about a shift in thinking regarding early diagnosis. There is no methodological standard for determining the appropriate cut-off value of a test’s accuracy to make a diagnosis because every test varies. One way the decision is made is by using Youden’s index method plotted against the ROC curve. A value of one on Youden’s index indicates no false positive and no false negative results; in other words, a perfect test [25]. In our study, when the three tests (GMA, neuroimaging, and HINE) were pooled, Youden’s index occurred at 0.9890 on the ROC curve, which is very high for any test, but especially high for a diagnosis with no biomarker. To contextualise our findings, here are some published diagnostic accuracy rates in other clinical diagnoses: (a) MRI has a sensitivity of 96% for detecting acute appendicitis [26]; (b) natriuretic peptides have a sensitivity of 95% for diagnosing acute heart failure [27]; (c) pathogenic tests have a sensitivity of 81% for detecting Parkinson Disease [28]; and (d) the Mini Mental State Examination has a sensitivity of 76% for detecting dementia [29]. Our pooled findings mean that, if clinicians use the combination of the GMA, imaging, and HINE and all three tests are indicating the infant has CP, they can be very confident that the child really does have CP and that a false positive diagnosis is highly unlikely. Like previous studies, we also found that over half the children with a long-term diagnosis of mild disability had a poor repertoire GMA score in the writhing period, indicating early neurological abnormalities [30]. An early diagnosis enables early intervention, which has important implications for optimising the child’s motor and cognitive outcomes [31,32], preventing secondary complications [33], and protecting parental mental health [34]. Early diagnosis is also having an important impact on the research field, enabling larger well-powered early intervention trials to be conducted, such as early motor training interventions (ACTRN12615000180516; ACTRN12617000006347). The emergent trial results, from Morgan using an intervention called “GAME” and Eliasson using a baby-friendly Constraint Induced Movement Therapy, appear to indicate that infants who have early intensive motor training intervention have a lower burden of disability. Furthermore, regenerative medicine agents are now being tested early, such as erythropoietin (ACTRN12614000669695), and stem cells (NCT02612155). Findings from these trials and others like them may have an important impact on children with CP’s long-term outcomes, and thus spur on the field to adopt early diagnosis as the new standard of care.

Our study has a number of limitations. First, due to the retrospective nature of our case-control study, there are likely changes in clinical care over time (especially neuroimaging quality), which may affect the accuracy of the results and the generalisability to contemporary care. Second, our cases and controls were recruited exclusively from hospitals, which will have higher rates of CP and mild disability than the general population, meaning selection bias is likely to have occurred. Third, the individuals selected as controls may under-represent the population that produced the cases because of the data collection method, again, meaning that selection bias may have occurred. Fourth, we used highly skilled masked assessors (e.g., GM Trust assessors and imaging experts) whose accuracy in scoring these tests may be higher than the general clinician population, affecting the generalisability of the results. Fifth, the GMA, imaging and HINE do not have perfect interrater reliability, and accordingly, our masked evaluators did not always agree on the scoring of each of the three tests. In spite of these methodological limitations and the lack of a biomarker, these retrospective observational study data can still be considered the best-available evidence for a diagnostic work-up that is likely to yield an early and accurate diagnosis of CP.

Future research into early diagnosis would be beneficial in understanding the long-term outcomes of children with only two of the three tests that are in the abnormal range, and conversely one test in the normal range. Preliminary indications from clinical trials suggest that these children almost never have a normal outcome, but often have a neurological diagnosis other than CP [31,32].

In conclusion, the clinical diagnosis of CP can be made early at three-months corrected age, with an accuracy of greater than 97%, when the child has absent fidgety general movements, a Hammersmith Infant Neurological Score below 57, and abnormal neuroimaging in the motor areas.

## Figures and Tables

**Table 1:** Characteristics of sample.

Infant Characteristics	Normal *n* = 147	Mild Disability *n* = 147	Cerebral Palsy *n* = 147
Gestational Age			
<32 weeks	44	42	44
32–36 weeks	63	65	63
>37 weeks	40	40	40
Gender (female %)	76 (52%)	75 (51%)	70 (48%)
Birthweight (g) mean (SD)	2082 (788)	2227 (813)	2109 (755)
Birth years			
2003–2006	119 (81%)	118 (80.5%)	128 (87%)
2007–2011	13 (9%)	18 (12%)	10 (7%)
2012–2016	15 (10%)	11 (7.5%)	9 (6%)
GMFCS level (*n* %)	-	-	
GMFCS I			55 (37.4%)
GMFCS II			36 (24.5%)
GMFCS III			26 (17.7%)
GMFCS IV			15 (10.2%)
GMFCS V			15 (10.2%)

MFCS, gross motor function classification system.

**Table 2:** Distribution of test results across 3 groups.

Assessments	Normal	Mild Disability	Cerebral Palsy
Writhing period GMs (*n*) (mean age 44 weeks)			
Normal	121(82%)	64 (44%)	1 (<1%)
Poor Repertoire	26 (18%)	83 (56%)	59 (40%)
Cramped Synchronised	0	0	85 (58%)
Chaotic	0	0	2 (1%)
Fidgety period GMs (*n*) (mean age 12 weeks CA)			
Normal	147 (100%)	104 (71%)	7 (5%)
Abnormal	0	36 (24%)	6 (4%)
Absent	0	7 (5%)	134 (91%)
HINE score at 3 months			
Mean (SD)	61 (6)	56 (6)	44 (12)
Median	63	57	46
Neuroimaging (*n*)			
Predict CP	2	2	116
Predict no CP	107	121	3
Unclear	38	24	27

GMs, general movements; CA, corrected age; HINE, hammersmith infant neurological examination; CP, cerebral palsy.

**Table 3:** Logistic regression of all tests.

Test	Odds Ratio	95% CI	Log Likelihood	Area under The ROC Curve	AIC—Statistical Measure
Hine 3 (continuous)	0.8	0.8–0.9	−183.6	85.07%	0.842
* Imaging (Unclear)	33.1	9.7–112.7	−88.2	96.00%	0.414
* Imaging (CP)	2204.0	485.2–10012.3			
Fidgety (abnormal removed)	686.4	235.8–1998.0	−60.0	96.16%	0.311
Multiple model					
Hine continuous (3 months)	0.9	0.8–1.0			
* Imaging (Unclear)	26.9	3.7–197.6			
* Imaging (CP)	2337.7	139.9–39074.3			
GMA (Absent fidgety vs. fidgety)	188.5	27.6–1288.5	−23.5	99.52%	0.143

* Imaging reference category: No CP. CI = confidence interval; ROC = receiver operating characteristic; AIC = akaike information criterion; CP = cerebral palsy; GMA = general movements assessment.
